# Structure of gut microbiota and characteristics of fecal metabolites in patients with lung cancer

**DOI:** 10.3389/fcimb.2023.1170326

**Published:** 2023-07-27

**Authors:** Xingbing Lu, Li Xiong, Xi Zheng, Qiuju Yu, Yuling Xiao, Yi Xie

**Affiliations:** ^1^ Department of Laboratory Medicine, West China Hospital, Sichuan University, Chengdu, China; ^2^ Lung Cancer Center, West China Hospital, Sichuan University, Chengdu, China

**Keywords:** lung cancer, gut micro-biome, 16S rRNA sequencing, GC/LC–MS/MS, metabolites

## Abstract

**Objective:**

The gut micro-biome plays a pivotal role in the progression of lung cancer. However, the specific mechanisms by which the intestinal microbiota and its metabolites are involved in the lung cancer process remain unclear.

**Method:**

Stool samples from 52 patients with lung cancer and 29 healthy control individuals were collected and subjected to 16S rRNA gene amplification sequencing and non-targeted gas/liquid chromatography-mass spectrometry metabolomics analysis. Then microbiota, metabolites and potential signaling pathways that may play an important role in the disease were filtered.

**Results:**

Firmicutes, Clostridia, Bacteroidacea, Bacteroides, and Lachnospira showed a greater abundance in healthy controls. In contrast, the *Ruminococcus gnavus(R.gnavus)* was significantly upregulated in lung cancer patients. In this respect, the micro-biome of the squamous cell carcinoma(SCC)group demonstrated a relatively higher abundance of Proteobacteria, Gammaproteobacteria, Bacteroides,and Enterobacteriaceae, as well as higher abundances of Fusicatenibacter and Roseburia in adenocarcinoma(ADC) group. Metabolomic analysis showed significant alterations in fecal metabolites including including quinic acid, 3-hydroxybenzoic acid,1-methylhydantoin,3,4-dihydroxydrocinnamic acid and 3,4-dihydroxybenzeneacetic acid were significantly altered in lung cancer patients. Additionally, the *R.gnavus* and Fusicatenibacter of lung cancer were associated with multiple metabolite levels.

**Conclusion:**

Our study provides essential guidance for a fundamental systematic and multilevel assessment of the contribution of gut micro-biome and their metabolites in lung cancer,which has great potential for understanding the pathogenesis of lung cancer and for better early prevention and targeted interventions.

## Highlights

- We combined multi-omics to find changes in the composition of the gut micro-biome in clinical lung cancer patients, characterized by a decrease in Probiotics and an overgrowth of potentially pathogenic bacteria.
- We are the first to study the relationship between lung cancer and the gut microbiota and its metabolite population, supporting the hypothesis that changes in specific microbiota influence the development of lung cancer. We suggest that decreasing levels of Firmicutes and Lachnospira and increasing levels of *Ruminococcus gnavus* group are associated with lung cancer.
- We found that most metabolites were closely associated with the characteristic micro-biome and that metabolism is attenuated in lung cancer patients, such as quinic acid, 3-hydroxybenzoic acid,1-methylhydantoin,3,4-dihydroxydrocinnamic acid and 3,4-dihydroxybenzeneacetic acid, and the five metabolites most closely associated with lung cancer showed a decreasing trend in lung cancer patients.

## 1 Introduction

Lung cancer(LC) is one of the most common malignancies worldwide, with increasing incidence and mortality rates worldwide, and will have 2.2 million new cases and 1.8 million deaths in 2020 (Sung et al., 2021). Eighty-five percent of LC pathological types are non-small cell lung carcinoma (NSCLC), including squamous cell carcinoma (SCC) and adenocarcinoma (ADC) (Majem et al., 2020). The vast majority of LC patients diagnosed at middle or advanced stages account for the poor prognosis and high mortality in this patient population (Zheng et al., 2020). China is a region with a high incidence of LC and increasing mortality, representing the main cause of high cancer deaths in both men and women (Chen et al., 2016). The significance of the human gut micro-biome is gradually being recognized. How to sustain a healthy gut micro-biome is becoming a growing concern.Over the years, the gut micro-biome as a symbiotic micro-biome has been widely studied and explored for its effects on immune regulation, substance metabolism, angiogenesis, the immune micro-environment, invasion and apoptosis in cancer (Geller and Straussman, 2018; Liu et al., 2020). Growing evidence substantiates that gut micro-biome is associated with many diseases, such as gastric cancer (Ravegnini et al., 2020), colorectal cancer (Mori et al., 2018), and breast cancer (Okubo et al., 2020).

Due to technical constraints and lack of adequate clinical data, the molecular patterns associated with microorganisms and the specific mechanisms by which bacterial metabolites drive cancer are not entirely clarified.Currently, limited studies of the gut micro-biome in NSCLC patients have been conducted. Zhuang et al. analyzed the gut micro-biome of 30 LC patients and found a significant correlation between bacteria such as Enterococcus and LC (Zhuang et al., 2019). Zheng et al. (2020) analyzed the gut micro-biome of patients with early LC, revealing the characteristics of the micro-biome and establishing a predictive model for the early diagnosis of LC. Although the “gut-microbiota-lung axis” (Liu et al., 2020) has been hypothesized and extensively studied, the specific mechanisms are not yet clear. The importance of microbiota-related metabolites in the development of LC suggests that it is necessary to study the role of the gut micro-biome and its metabolome in the pathogenesis of LC. To date, it remains unclear how and which interactions of the micro-biome and metabolites promote LC development. Evidence for the relevance of gut micro-biome characteristics and their metabolites with LC is limited, and potential molecular mechanisms need to be further investigated. Therefore, we aimed to identify changes in the gut micro-biome and its metabolites in LC patients, to better understand the involvement of the gut micro-biome in the pathogenesis of LC, and to explore new features of LC progression, which are essential for early diagnosis and prevention of LC.

## 2 Materials and methods

### 2.1 Study participants

A total of 81 fecal samples were collected from 52 LC patients (median age: 56.9 years old) and 29 matched HC individuals from West China Hospital, Sichuan University (**Table 1**). All patients were diagnosed with LC for the first time based on their histopathological features, and were classified as malignant tumors by TNM after surgery. The LC patients and healthy controls with the following conditions were excluded: congestive cardiac failure, respiratory failure, intestinal disease, renal failure, severe liver dysfunction, or consumption of probiotics or antibiotics within one month before specimen collection. In addition, all patients included in our study were all diagnosed with LC for the first time, they did not receive any prior drug treatment directly related to their LC diagnosis, and they did not have any other comorbid oncological disease. The clinical characteristics of all participants are listed in **Table 1**, and there were no significant differences in age, body mass index (BMI) and male-female ratio between the two groups (P>0.05). Each patient signed an informed consent form before the study. The protocol was approved by the Ethics Committee of the West China Hospital, Sichuan University.

**Table 1:** Baseline characteristics of the discovery cohort.

Chacracteristic	Patients with lung cancer(n=52)	Haelth Control (n = 29)	P-value
Demographics/anthropometric			
Age year (mean ± SD)	56.92±12.37	50.72±16.54	0.060
Male/female (No.)	36/16	16/13	0.206
BMI (kg/m2) (mean ± SD)	22.86±2.30	22.93±2.74	0.892
Tumor type (%)			
ADC	30(57.69%)	N/A	
SCC	22(42.31%)	N/A	
Tumor stage(%)			
I	20(38.46%)	N/A	
II	9(17.31%)	N/A	
III	15(28.84%)	N/A	
IV	8 (15.39%)	N/A	

TNM, tumor node metastasis scale.

Unpaired t-test was used to compare age and BMI between lung cancer group and healthy controls; Fisher’s exact test was used to compare gender distribution between the two groups. N/A, not applicable.

### 2.2 DNA extraction and PCR amplification

Total DNA extraction from the 81 fecal specimens was conducted according to the instructions of the DNeasy PowerSoil kit (Qiagen, Hilden, Germany). DNA concentration and purity were determined using NanoDrop2000 (Thermo Fisher Scientific, Waltham, MA, USA), and the quality of the extracted DNA was inspected by 1% agarose gel electrophoresis. PCR amplification of the V3-V4 variable region was performed using343F(5′-TACGGRAGGCAGCAG-3′) and 798R(5′-AGGGTATCTAATCCT-3′) (Nossa et al., 2010) primers by the following amplification procedure: pre-denaturation at 95°C for 3 min, 27 cycles (denaturation at 95°C for 30 s, annealing at 55°C for 30 s, extension at 72°C for 30 s), and extension at 72°C for 10 min.

### 2.3 Library construction and sequencing

The Amplicon quality was visualized using agarose gel electrophoresis. The PCR products purified with AMPure XP beads (Agencourt) and amplified for another round of PCR. After purified with the AMPure XP beads again, the final amplicon was quantified using Qubit dsDNA Assay Kit (Thermo Fisher Scientific,USA). The concentrations were then adjusted for sequencing. Sequencing was performed on an Illumina NovaSeq 6000 with 250 bp paired-end reads. (Illumina Inc., San Diego, CA; OE Biotech Company; Shanghai, China).

### 2.4 Sample preparation for metabolome profiling

Sixty milligrams of feces from each sample were added to 360 μL of precooled methanol and 40 μL of internal standard (L-2-chloro-phenylalanine, 0.3 mg/mL; methanol configuration). The mixture was ground, stirred, incubated and centrifuged. The supernatant was concentrated, dried, dissolved and resuspended for subsequent GC-MS/MS analysis. Similarly, 60 mg of feces from each sample was added to 650 μL of methanol-water (V:V=4:1) and 20 μL of internal standard. The mixture was ground, stirred, incubated and centrifuged. The supernatant was filtered and transferred for LC-MS/MS analysis.

### 2.5 Bioinformatic analysis

#### 2.5.1 16SrRNA amplicon sequencing analysis process

The library sequencing and data processing were conducted by OE biotech Co., Ltd. (Shanghai, China). Raw sequencing data were in FASTQ format. Paired-end reads were then preprocessed using Cutadapt software to detect and cut off the adapter. After trimming, paired-end reads were filtering low quality sequences, denoised, merged and detect and cut off the chimera reads using DADA2 (Callahan et al., 2016) with the default parameters of QIIME2 (Bolyen et al., 2019) (2020.11). At last, the software output the representative reads and the ASV abundance table. The representative read of each ASV was selected using QIIME2 package. All representative reads were annotated and blasted against Silva database (Version 138) using q2-feature-classifier with the default parameters.

QIIME2 software was used for alpha and beta diversity analysis. Alpha diversity including Chao1 index and Shannon index was used to estimate the diversity of the micro-biome in the sample (Hill et al., 2003), and then statistical t-test was used to detect if there was a significant difference in the index values between the two groups. Beta diversity analysis represents a comparison of micro-biome community composition and is used here to assess differences between microbial community composition (Zhuang et al., 2019). The basic output of this comparison is a distance matrix that represents the difference between every two samples in the community. The binary jaccard distance matrix performed by R package was used for binary jaccard Principal coordinates analysis (PCoA) to estimate the beta diversity. Then the R package was used to analyze the significant differences between different groups using ANOVA/Kruskal Wallis/T test/Wilcoxon statistical test.

LEfSe is a software package for discovering high-dimensional biomarkers with inputs that include genes,metabolites, and classification.We first used the non-parametric factorial Kruskal-Wallis (KW) sum-rank test to detect specificspecies relating significant abundance differences in two groups (Zhuang et al., 2019). We then estimated the effect of each component (species) by LEfSe linear discriminant analysis (LDA). In order to detect the species contributing to the abundance differences in different groups of microbial communities,we carried out a test of significance differences between groups. Based on the obtained community abundance data, rigorous statistical methods were used to detect species with different richness in different groups (samples) of microbial communities, and hypothesis testing was performed to assess the significance of these observed differences (Zhuang et al., 2019).

### 2.6 GC/MS and LC-MS data preprocessing and statistical analysis

The obtained GC/MS raw data in D format were transferred to.abf format via software Analysis Base File Converter for quick retrieval of data. Then, data were imported into software MS-DIAL, which performs peak detection, peak identification, MS2Dec deconvolution, characterization, peak alignment, wave filtering, and missing value interpolation. Metabolite characterization was based on LUG database. A data matrix was derived. The three-dimensional matrix includes: sample information, the name of the peak of each substance, retention time, retention index, mass-to-charge ratio, and signal intensity. In each sample, all peak signal intensities were segmented and normalized according to the internal standards with RSD greater than 0.1 after screening. After the data was normalized, redundancy removal and peak merging were conducted to obtain the data matrix.

The original LC-MS data were processed by software Progenesis QI V2.3 (Nonlinear, Dynamics, Newcastle, UK) for baseline filtering, peak identification, integral, retention time correction, peak alignment, and normalization. Main parameters of 5 ppm precursor tolerance, 10 ppm product tolerance, and 5% product ion threshold were applied. Compound identification were based on precise mass-to-charge ratio (M/z), secondary fragments, and isotopic distribution using The Human Metabolome Database (HMDB), Lipidmaps (V2.3), Metlin, and self-built databases. The extracted data were then further processed by removing any peaks with a missing value (ion intensity = 0) in more than 50% in groups, by replacing zero value by half of the minimum value, and by screening according to the qualitative results of the compound. Compounds with resulting scores below 36 (out of 60) points were also deemed to be inaccurate and removed. A data matrix was combined from the positive and negative ion data.

The matrix was imported in R to carry out Principle Component Analysis (PCA) to observe the overall distribution among the samples and the stability of the whole analysis process. Orthogonal Partial Least-Squares-Discriminant Analysis (OPLS-DA) and Partial Least-Squares-Discriminant Analysis (PLS-DA) were carried out to visualize the metabolic alterations between NSCLC patients and healthy controls after mean centering and unit variance scaling. To prevent overfitting, 7-fold cross-validation and 200 Response Permutation Testing (RPT) were used to evaluate the quality of the model. The variable importance in projection values and S-plots were selected to obtain the significant variables for subsequent analysis.Student’s t-test was used to measure the significance of each variable among those selected. Differential metabolites found from the OPLS-DA models were identified or tentatively annotated. Differential metabolites analysis were conducted using the R package MetaboAnalystR. The significantly altered metabolites were determined by variable importance in projection (VIP) scores from pairwise PLS-DA analysis and pairwise comparisons using the Wilcoxon rank-sum test. Meanwhile, the differential metabolites with VIP value >1.0 and P value <0.05 and FC value <0.5 or >2 were selected. Interactions among disease associated metabolites were estimated by Spearman’s rank correlation. Metabolite set enrichment analysis (MSEA) was performed using the online tool MetaboAnalyst. All heat maps were drawn using the R package Complex Heatmap.

### 2.7 Statistical analysis

All statistical calculations were performed in R3.4.3.The correction of the P-value is responsible for the false discovery rate (FDR). Age and BMI data were expressed as the mean ± standard deviation (SD), and differences between groups were assessed using a one-way analysis of variance (ANOVA). Inter-group comparisons were performed using aWilcoxon rank sum test of non-parametric data. A t-test was applied after the results were reflected as visual metrics using a histogram. Multiple clusters were analyzed using the nonparametric factor Kruskal-WallisWallis. Fisher’s exact test was performed on categorical variables. The dissimilarity tests among groups(PERMANOVA) were conducted on Euclidean distance for metabolites and Bray-Curtis distance for bacteria,with10,000 permutations in the R package, vegan. A P-value below 0.05 was considered statistically significant.

## 3 Results

### 3.1 Gut micro-biome profile of lung cancer patients

**Figure 1:**
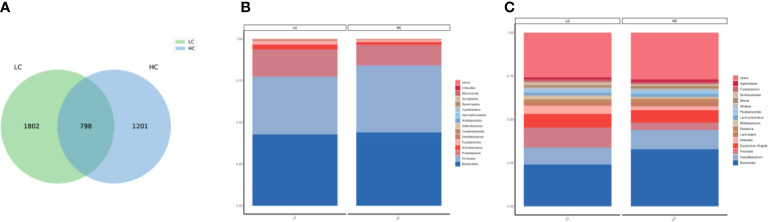
**(A)** Venn diagam of the lung cancer and healthy control groups. **(B)** Community composition histogram of LC and HC at phylum level. **(C)** Community composition histogram of LC and HC at genus level.

According to the Venn diagram, the LC group and the healthy group contained core 798 ASVs, and 1082 ASVs and 1201 ASVs were unique to the LC and HC, respectively (**Figure 1A**). At the phylum level, Bacteroidota,Firmicutes,Proteobacteria and Actinobacteriota accounted for the major components of the gut microbiota (**Figure 1B**). The FBR (ratio of Firmicutes to Bacteroidetes) was calculated as 0.810 (0.346/0.427) and 0.913 (0.401/0.439) based on the percentage of Firmicutes and Bacteroidetes in LC patients and HC, respectively. At the genus level, the most common in LC and HC were Bacteroides (**Figure 1C**).

### 3.2 The alpha diversity and beta diversity of the gut micro-biome

**Figure 2:**
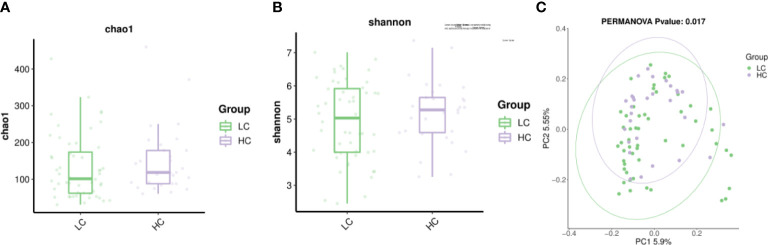
The alpha diversity include Chao1 index and Shannon index. **(A)** Chao1 indicates species richness between the two groups. **(B)** Shannon indicates species evenness between the two groups. **(C)** β-diversity (expressed by principal coordinate analysis, PCoA) was significantly different between LC patients and healthy controls.

The alpha diversity of the gut micro-biome in the LC group and HC population was similar in Chao1 and Shannon (**Figures 2A, B**). The beta diversity of LC patients was significantly different from HC (p=0.017) as assessed by principal coordinate analysis (PCoA) (**Figure 2C**).

### 3.3 Specific gut micro-biome signatures in lung cancer patients

**Figure 3:**
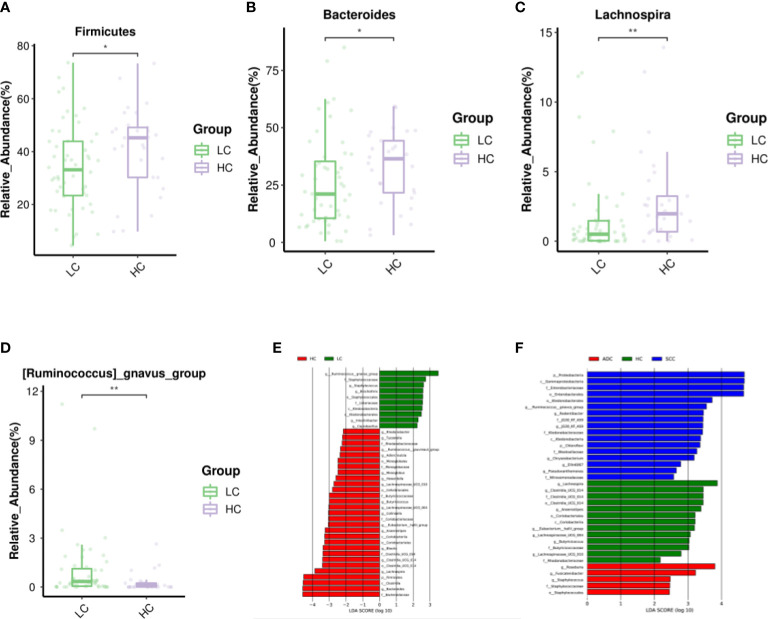
Differential abundance of gut microbiota in LC and HC. The taxa decreased **(A–C)** and increased **(D)** in LC patients at the phylum, and genus, p<0.05. Purple and Green represented the HC(n=29) and LC patients(n=52), respectively.Starred samples (*/**) were used to demonstrate the significant difference between the group. **(E)** Discriminant analysis of LEfSe species difference between LCand HC. **(F)** Discriminant analysis of LEfSe species difference between SCC, ADC, and HC. The LDA score obtained by linear regression analysis (LDA), the larger the LDA score, the greater the influence of species abundance on the difference effect. Different colors indicate different groups. Red bars indicate species with relatively high abundance in the red group. Green bars indicate species with relatively high abundance in the green group. Blue bars indicate species with relatively high abundance in the blue group.

The LEfSe study showed that the number of micro-biome promoting healthy, such as Firmicutes, Clostridia, Bacteroidaceae, Bacteroides and Lachnospira, was also significantly reduced in LC patients. In contrast, *Ruminococcus gnavus(R.gnavus)* was higher in patients with LC and SCC sub-types than in HC patients (**Figure 3**). In addition, the major sub-types of LC appear to have different microbiota profiles.For example,Proteobacteria,Gammaproteobacteria, Enterobacterales and Enterobacteriaceae showed higher abundance in SCC patients, while Roseburia and Fusicatenibacter were more abundant in ADC (**Figures 3**, **S1**).

### 3.4 Metabolomic signature of gut samples in HC and LC patients

**Figure 4:**
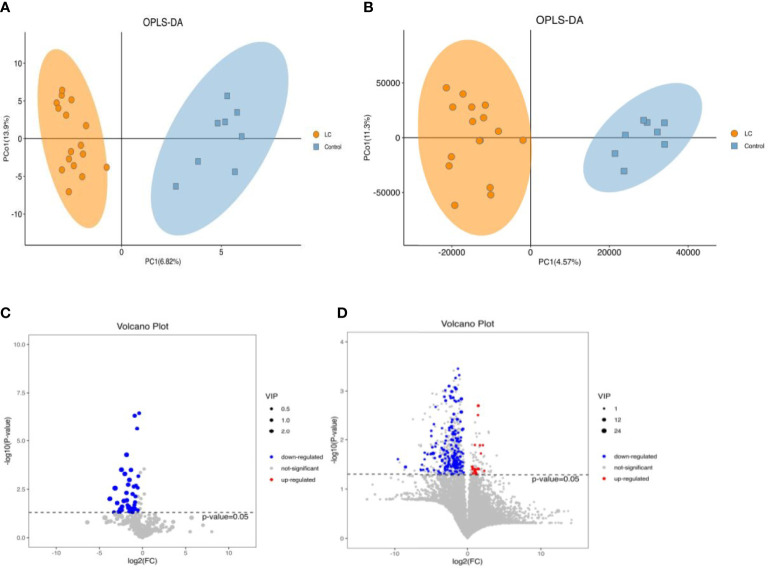
The alterations in fecal metabolites of LC and HC (Left,GC-MS; Right,LC-MS). **(A, B)** Scatter plotof OPLS-DA model of LC and HC. **(C, D)** Volcano plots for LC and healthy group. P<0.05 for significant difference. Red dots represent those up-regulated, blue dots represent those down-regulated.

Eight stool samples with the highest colony abundance in each group were selected for GC/LC-MS untargeted metabolic analysis. permutation plots indicated that the fit (R2Y) and predictive ability (Q2Y) of the model were 0.933/0.914 and 0.264/0.217, respectively, indicating good fit and predictive ability of the OPLS-DA model(**Figures S2A, S2B**). The OPLS-DA plots with supervised analysis function fully reflected the significant differences between metabolites of LC and HC groups, and the model was valid and not over-fitted (**Figures 4A, B**). Variable importance (VIP) values obtained from the OPLS-DA model results were used to rank the overall contribution of each variable to group differentiation. In our study, the default screening criteria for differential metabolites were set to VIP>1 and P<0.05, plotted as volcano plots and differential metabolite heat maps (**Figure S2C, D**). Volcano plots showed that almost all GC-MS differential metabolites were down-regulated in LC patients (**Figure 4C**) and only a few LC-MS differential metabolites were up-regulated (**Figure 4D**).

### 3.5 Potential of metabolic biomarkers

**Figure 5:**
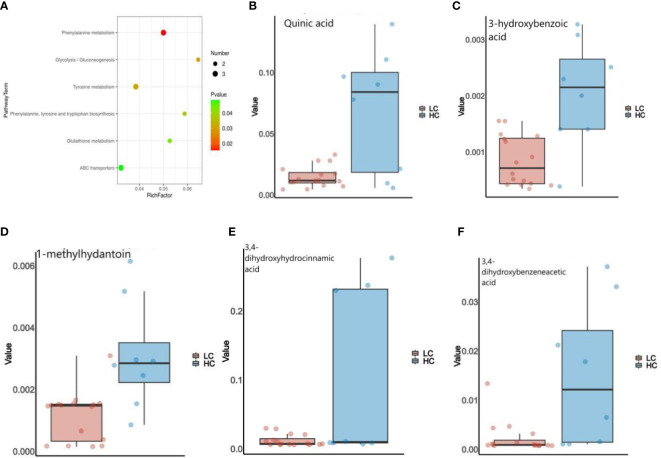
**(A)** Enrichment analysis of the GC&LC-MS/MS metabolites in feces samples of LC and HC. **(B–F)** The Wilcoxon test showed that five fecal metabolites were significantly altered in LC patients.

In addition, We used KEGG annotation-based metabolic enrichment and pathway analysis, employing a hypergeometric distribution test to map LC patients’ altered metabolites to their biochemical pathways.Differential metabolites appear to be heavily involved in amino acid metabolism, including tyrosine metabolism,phenylalanine metabolism,and phenylalanine, tyrosine, and tryptophan biosynthesis in LC patients compared to the HC group (**Figure 5A**). From here, we screened five intestinal differential metabolites with the highest relevance to LC patients, which were quinic acid, 3-hydroxybenzoic acid, 1-methylhydantoin, 3,4-Dihydroxyhydrocinnamic acid and 3,4-Dihydroxybenzeneacetic acid, all of them showed a down-regulated state, significantly lower than the HC group. (**Figures 5B–F**; **Table S1**).

For the different sub-types of LC, differential metabolites are closely associated with steroid biosynthesis, caffeine metabolism and amino acid metabolism mainly in ADC patients (**Figure S3A**). Tyramine levels were upregulated in the ADC group, but 1,7-dimethyluric acid, stigmasterol and 3-hydroxybenzoic acid exhibited downregulation (**Table S2**). In the SCC group, differential metabolites were mainly associated with citrate cycle (TCA cycle), central carbon metabolism in cancer, and glyoxylate and dicarboxylate metabolism(**Figure S3B**). 3β-Hydroxycholest-5-en-26-oic acid and stearic acid showed up-regulation in the SCC group, but aconitic acid,behenic acid,and glucose-6-phosphate were down-regulated (**Table S3**).Furthermore, the differential metabolites in ADC patients relative to SCC are mainly involved in ABC transporters and carbon metabolism in cancer is closely related (**Figure S3C**). In addition, D-glucose and maltotriose were upregulated in the ADC group,but stearic acid, Udp-n-acetylglucosamine and palmitic acid expression were down-regulated (**Table S4**).

### 3.6 Correlation between the altered metabolites and gut micro-biome

Considering the intimate contact between gut microbes and metabolites, we appraised the reliability of correlations between altered taxa and LC-related metabolites (**Figures S4A, B**). We used the specific gut micro-biome obtained from the LEfSe analysis of LC patients as a baseline, and the software set the correlation coefficient at 0.5 as a threshold and plotted the network of relationships with fecal metabolites.From the network diagram, we found a negative correlation between the LC characteristic bacterium *R.gnavus* and most metabolites, while a positive correlation between the probiotic Lachnospira, Firmicutes and ADC subtype characteristic bacterium Fusicatenibacter and most metabolites. For example, picolinic acid was positively correlated with Lachnospira, Firmicutes and Fusicatenibacter, while it was negatively correlated with *R. gnavus* (P<0.05, r=0.5) (**Figure S4C**).

## 4 Discussion

An increasing body of evidence suggests that gut dysbiosis can cause immune dysregulation and chronic inflammation and even promote the progression of various tumors, including LC, through the ‘gut-lung’ axis (Wang et al., 2020; Ge et al., 2021; Zhao et al., 2021). Our study showed a lower proportion of Firmicutes and a higher proportion of Proteobacteria and Actinobacteriota in LC patients, which is inconsistent with the results reported by Qian et al. (2022) (**Figure 1B**). Overall, we found differences in the composition of the gut micro-biome between the LC patients and the HC group, characterized by loss of probiotics and overgrowth of potentially pathogenic bacteria.Interestingly, the ratio of Firmicutes to Bacteroidetes (FBR) has been used as an indicator of metabolism, nutrition and evaluation of gut micro-biome in cancer patients (Xi et al., 2022). Reduced FBR may be associated with the progression and recurrence of breast cancer (Okubo et al., 2020) and colorectal cancer (Gao et al., 2017). In the present study, there was little difference in FBR in LC patients, and there is insufficient evidence to speculate whether FBR is directly related to LC disease. In our study, the LC and healthy groups did not differ in the alpha diversity of the gut micro-biome and were highly similar (**Figure 1**). Researches found that high diversity increased circulating memory CD8 T cells and natural killercells and prolonged progression-free survival in cancer patients (Bernicker and Quigley, 2019). Conversely, low diversity can cause a decrease in host immunity and an increase in oncogenic metabolites that induce and promote tumor progression (Wang et al., 2011).

We characterized the gut micro-biome of LC patients from LefSe and histograms and identified microbiota that may contribute to the development of LC. At the phylum level, Firmicutes are significantly enriched in the HC, containing a large number of commensal species that are part of the healthy human micro-biome. Firmicutes are known to promote single-chain fatty acids(SCFAs) production in the colonic lumen and to regulate inflammation and tumor formation, which may be associated with reduced abundance of this bacterium in LC (Sivan et al., 2015). Studies have confirmed that Firmicutes and Bacteroidota (Bacteroidetes) catabolize carbohydrates in the colon to produce SCFAs,of which Firmicutes are the main butyrate producers and Bacteroidetes mainly produce acetate and propionate (Allers et al., 2020). SCFAs such as butyrate can inhibit the proliferation of LC cells by regulating the expression of p21, while propionate can inhibit cell growth by inducing apoptosis and cell cycle arrest (Mowday et al., 2022). In our study, Firmicutes family (including Clostridia,and Lachnospira) and Bacteroidota family (including Bacteroidaceae and Bacteroides) were significantly reduced in LC patients with diminished tumor suppressive effects and promoted LC development, in agreement with the conclusion reached by Xi et al. (Kim et al., 2019). The results of our study are also similar to those reported in healthy Korean subjects, confirming that the Bacteroidetes micro-biome plays a positive role in intestinal health and tumor prevention (Noh et al., 2021; Wu et al., 2021). It was found that the anti-inflammatory genus Lachnospira belongs to SCFA-producing bacteria and its abundance decreased significantly with increasing body weight (Mayengbam et al., 2019). Lachnospira levels have been found to be significantly reduced in patients with Alzheimer’s disease, presumably positively correlating with improvements in cognitive function (Guo et al., 2021). Studies have reported that *R.gnavus* inhibits TNF-α secretion and may suppress lung cancer metastasis (Biggs et al., 2017). In addition, *R.gnavus* was significantly associated with a low risk of Alzheimer’s disease and a high risk of amblyopic lateral sclerosis (Ning et al., 2022). This suggests that *R.gnavus* has different roles in different diseases. In the present study, *R.gnavus* showed a significantly high expression abundance in LC, especially in SCC patients. We suggest that elevated levels of *R.gnavus* are associated with LC, and we further reveal that impairment of normal gut micobiome function is associated with the progression of LC. We hope that the results of this study will provide some guidance for using gut microbes as biomarkers to assess the progression of LC or provide intervention targets to control the disease progression.

Furthermore, we found that patients with different pathological types of LC seem to have their own unique gut micro-biome that may be involved in the development of SCC and ADC subtypes. It is well known that the family Enterobacteriaceae belongs to the order Proteobacteria, Gammaproteobacteria and Enterobacterales, and that they show high abundance in SCC patients, similar to the results of Jain et al. (2019). Interestingly, Proteobacteria is a potential pathogen causing intestinal dysbiosis in LC patients (Liu et al., 2019) and can promote inflammation and carcinogenesis by activating Toll-like receptors or NOD-like receptors, producing genotoxins and virulence factors (Schwabe and Jobin, 2013; Bingula et al., 2020). Geller et al. showed that tumor bacteria in pancreatic cancer, especially Gammaproteobacteria, account for gemcitabine resistance and exacerbate cancer progression (Geller et al., 2017). Moreover, the presence of Gammaproteobacteria was found to correlate with low PD-L1 expression and poor response to checkpoint-based immunotherapy, translating into poor survival (Boesch et al., 2021). However, the detailed link between the enteropulmonary axis and lung immunity or cancer development remains to be further elucidated. Zhao et al. (2018) found that Roseburia and Fusicatenibacter are elevated in patients with Hashimoto’s thyroiditis and may be involved in disease progression. Roseburia belongs to the healthy human micro-biome and produces SCFAs, whose relative abundance is higher in patients with LC, and who are more likely to experience gastrointestinal reactions after chemotherapy (Zhang et al., 2020; Mammadova et al., 2021). The highest abundance of Fusicatenibacter and Roseburia in the ADC group in this study was similar to the results of Qian et al. (2022).

The decreased diversity of the gut micro-biome may be partly responsible for the apparent lack of intestinal metabolites that we observe in LC patients. There is evidence that gut micro-biome promote chronic inflammation and tumorigenesis, such as colorectal cancer, through their metabolites (Han et al., 2020). For example, *Granulicatella adiacens* in sputum promotes LC through polyamine metabolism (Cameron et al., 2017). We are the first to study LC in relation to the gut micro-biome and its metabolites. By non-targeted assay analysis, significant differences in micro-biome functional abundance and metabolites were observed between the two groups. Healthy controls had a significantly higher micro-biome functional profile, whereas the gut micro-biome of LC patients showed a decreasing trend in metabolic capacity during the disease.According to the screening criteria, we summarized that the top five metabolites with the best correlation to LC were quinic acid, 3-hydroxybenzoic acid, 1-methylhydantoin, 3,4-Dihydroxyhydrocinnamic acid and 3,4-Dihydroxybenzeneacetic acid (**Figure 5**; **Table S1**). It was discovered that quinic acid (QA) can only be synthesized indirectly by healthy gut micro-biome, such as Firmicutes, and has antioxidant, anti-inflammatory and anti-cancer effects (Candeias et al., 2018; Naranjo et al., 2018). QA enhances the therapeutic response to PD1(L1) in colon cancer (Lin et al., 2018), and which also inhibits p-PI3K and p-AKT expression and suppresses the proliferation and growth of melanoma cells (Kang et al., 2021). 3-hydroxybenzoic acid (3-HBA) has analgesic and anti-inflammatory effects, which is associated with lipid homeostasis regulation, and has not been studied in relation to tumors (Khan et al., 2016). Li et al. (2014) found that that 1-methylhydantoin (MH) inhibits the proliferation and induces apoptosis in colon cancer SW480 cells, while blocking the G0/G1 phase of the cell cycle. 3,4-Dihydroxyhydrocinnamic acid (also known as dihydrocaffeic acid, DHCA) is present in normal human body fluids and has antioxidant activity (Amic et al., 2018). Hydrogels containing DHCA components were found to release doxorubicin (DOX) and to be effective in killing colon tumor cells (Liang et al., 2019). 3,4-Dihydroxybenzeneacetic acid (also known as 3,4-dihydroxyphenylacetic acid, DOPAC) not only inhibits colon cancer cell proliferation (Catalan et al., 2020), but also reflects the progression of osteosarcoma (Bandala et al., 2021), and combines with Fe3O4@TiO2 nanocomposites to improve the sensitivity of tumor treatment (Liu et al., 2021). Interestingly, the probiotics Firmicutes and Lachnospira were positively associated with most metabolites in LC patients, whereas opportunistic pathogenic bacteria such as *R. gnavus* were negatively associated with most metabolites. Thus, the present study adequately confirmed that most metabolites were able to inhibit LC tumor cell proliferation and induce apoptosis, with the decrease of probiotics and increase of opportunistic pathogenic bacteria, resulting in the decrease of metabolism of most intestinal metabolites in LC patients, thus affecting the malignant progression of LC disease. Undoubtedly, changes in the micro-biome and its associated metabolites caused by dysbiosis of the gut micro-biome are key pathways in the development of LC, laying the foundation for early diagnosis and active prevention of LC.

## 5 Conclusion

In conclusion, this is the first study to link LC to the gut micro-biome and its metabolites. We determined that the gut micro-biome and its metabolites vary with the different stages of LC development. In addition, we preliminarily explored the possible relationship between gut micro-biome and intestinal metabolites in LC patients to provide some guidance for further assessment of LC progression or for intervention to control disease progression. However, several limitations were found in our study. First, a limited number of pathological types of LC were included in our study, and the sample size was relatively small. Moreover, there were differences in the genetic background, diet and environment of the experimentally recruited population. It should also be borne in mind that the limitations of 16S rRNA gene sequencing and GC/LC-MS methods affected the reliability of our findings to a certain extent. Indeed, additional LC tissue and plasma specimens for transcriptomic and metabolomic analysis are needed to validate and study the interactions between gut micro-biome and hosts.

## Data availability statement

The datasets presented in this study are available in NCBI Sequence Read Archive (SRA) with the accession number PRJNA882575.

## Ethics statement

Fecal samples were collected in this research without any medical expense or suffering by the subjects, and the results were used for scientific research purposes. We declare that none of the authors have any conflicts of interest or financial ties to disclose. This study was carried out in accordance with the recommendations of the Ethical Guidelines for Biomedical Research, West China Hospital, Sichuan University. The protocol was approved by the West China Hospital, Sichuan University Ethics Review Committee (reference: 2021-1120, 04/11/2021). All subjects gave written informed consent in accordance with the Declaration of Helsinki. The subjects' rights were adequately protected, and there was no potential risk to the subjects.

## Author contributions

XBL, YLX, and YX contributed to the conception and design of the study. XBL, LX, and XZ performed the experiment and statistical analysis. XBL wrote the first draft of the manuscript. LX and XZ wrote sections of the manuscript. QJY helped perform the analysis with constructive discussions. All authors contributed to manuscript revision and approved the submitted version.
