# Supplementary material for: Structure of gut microbiota and characteristics of fecal metabolites in patients with lung cancer
Source: Front Cell Infect Microbiol. 2023 Jul 27;13:1170326. doi: 10.3389/fcimb.2023.1170326 (PMC10415071; doi:10.3389/fcimb.2023.1170326)
Supplement: Supplementary file 1 [file DataSheet_1.docx]

Supplementary Material

**A B C D**

**
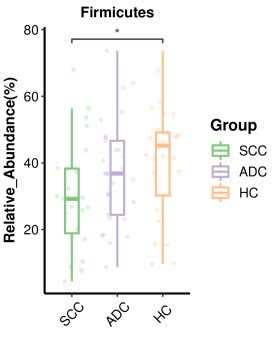

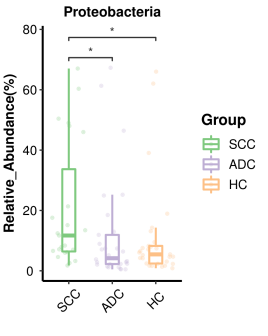

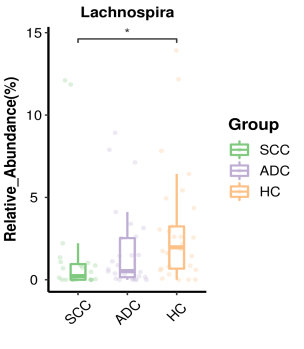

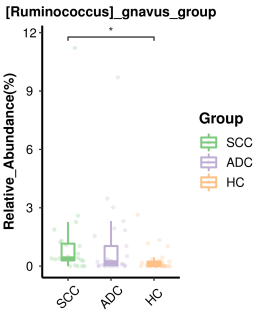
**

Figure S1|Differential abundance of gut microbiota in LC and HC. The taxa decreased (A,C) and increased (B,D) in patients with SCC lung cancer at the phylum, and genus, p <0.05. Green,Purple,and Orange represented the SCC and HC, respectively.Starred samples (*/**) were used to demonstrate the significant difference between the group

**A B
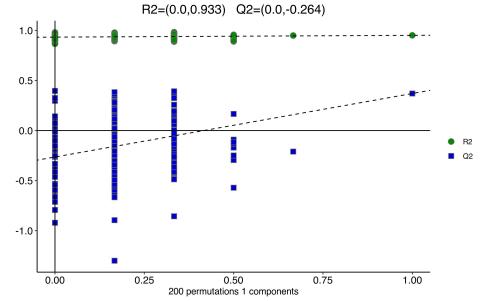

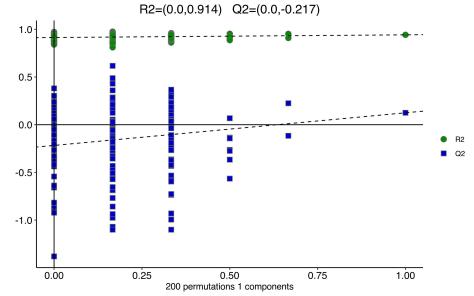
**

**C D
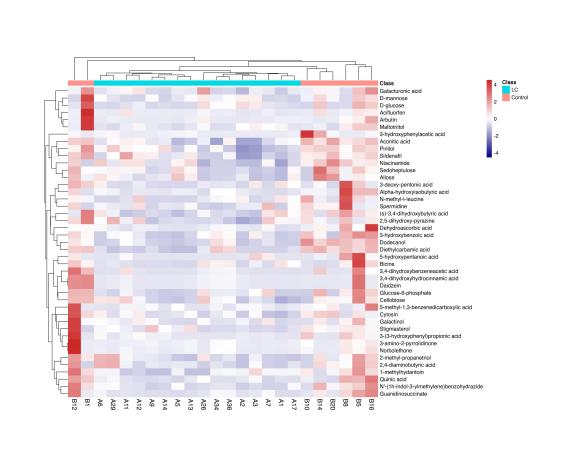

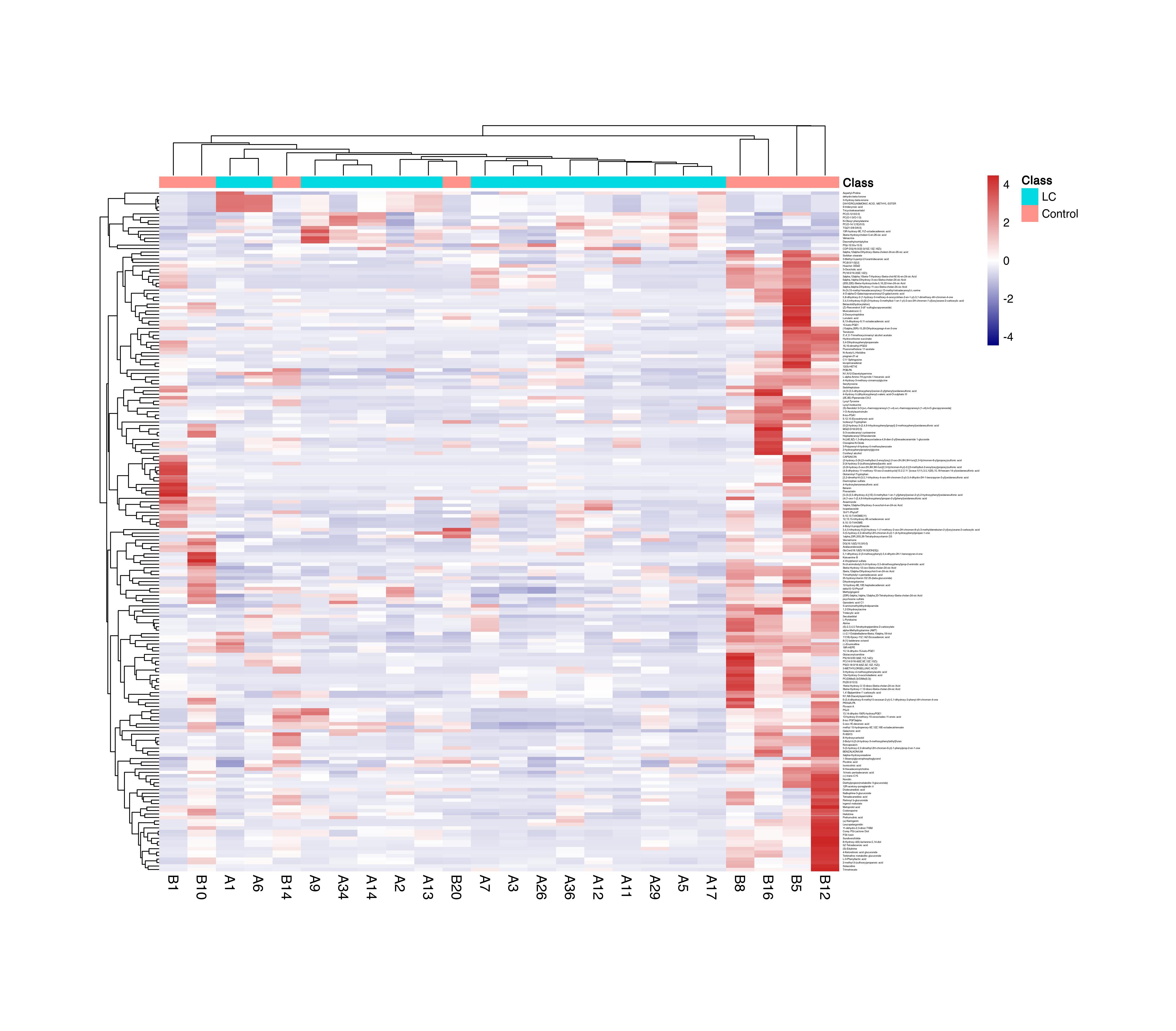
**

**Figure S2|**The alterations in fecal metabolites of LC patients（Left ,GC-MS;Right,LC-MS）.**(A-B)** Permutation test of OPLS-DA model for LC and healthy group. **(C-D)** Differential metabolite heat map of LC and HC.

A B C

**
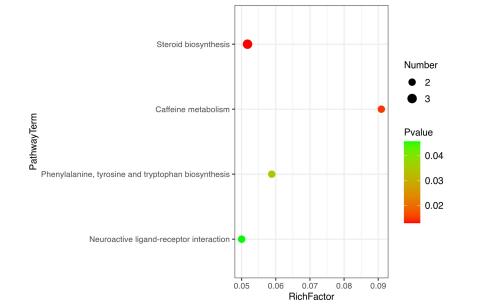

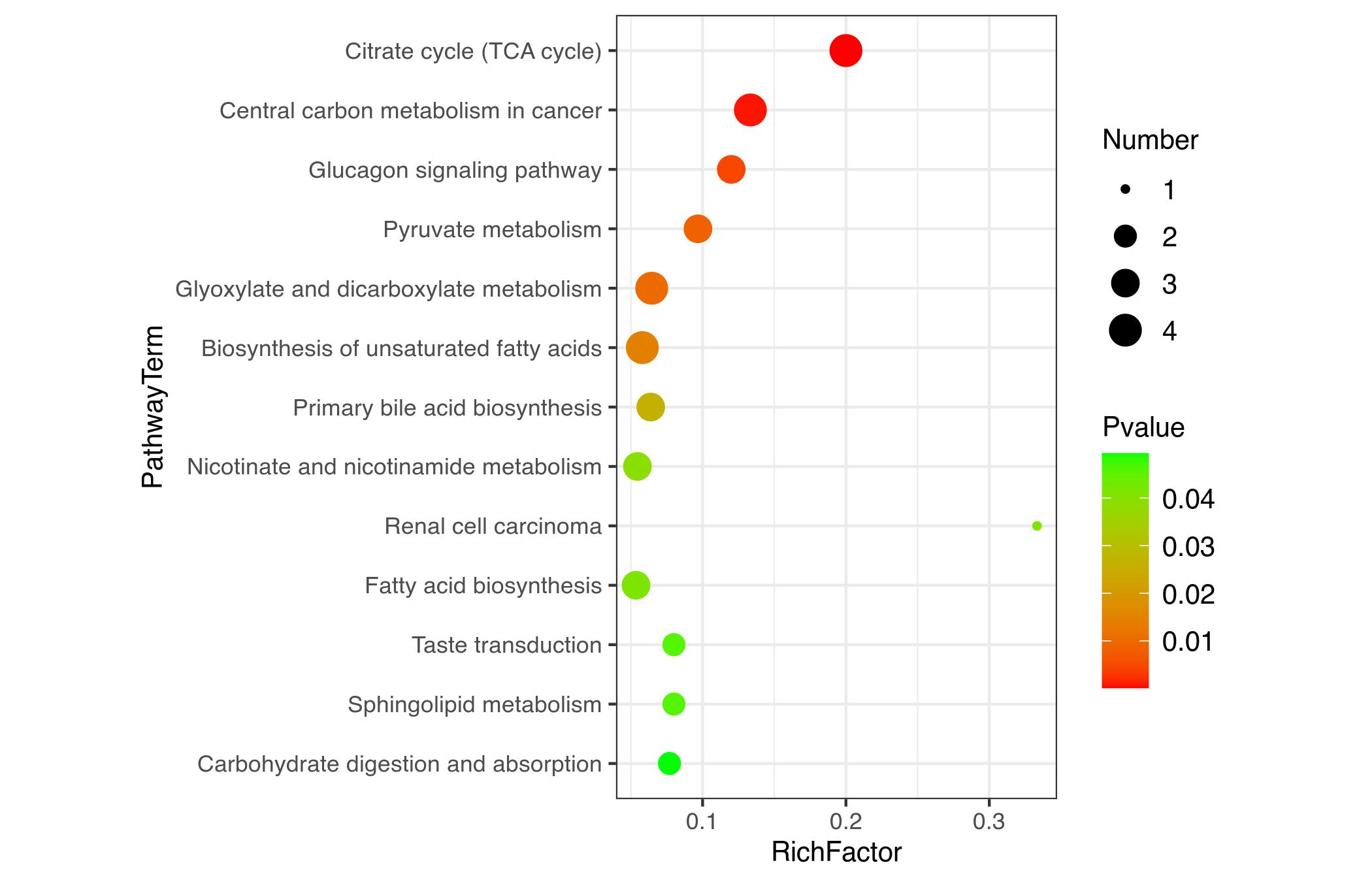

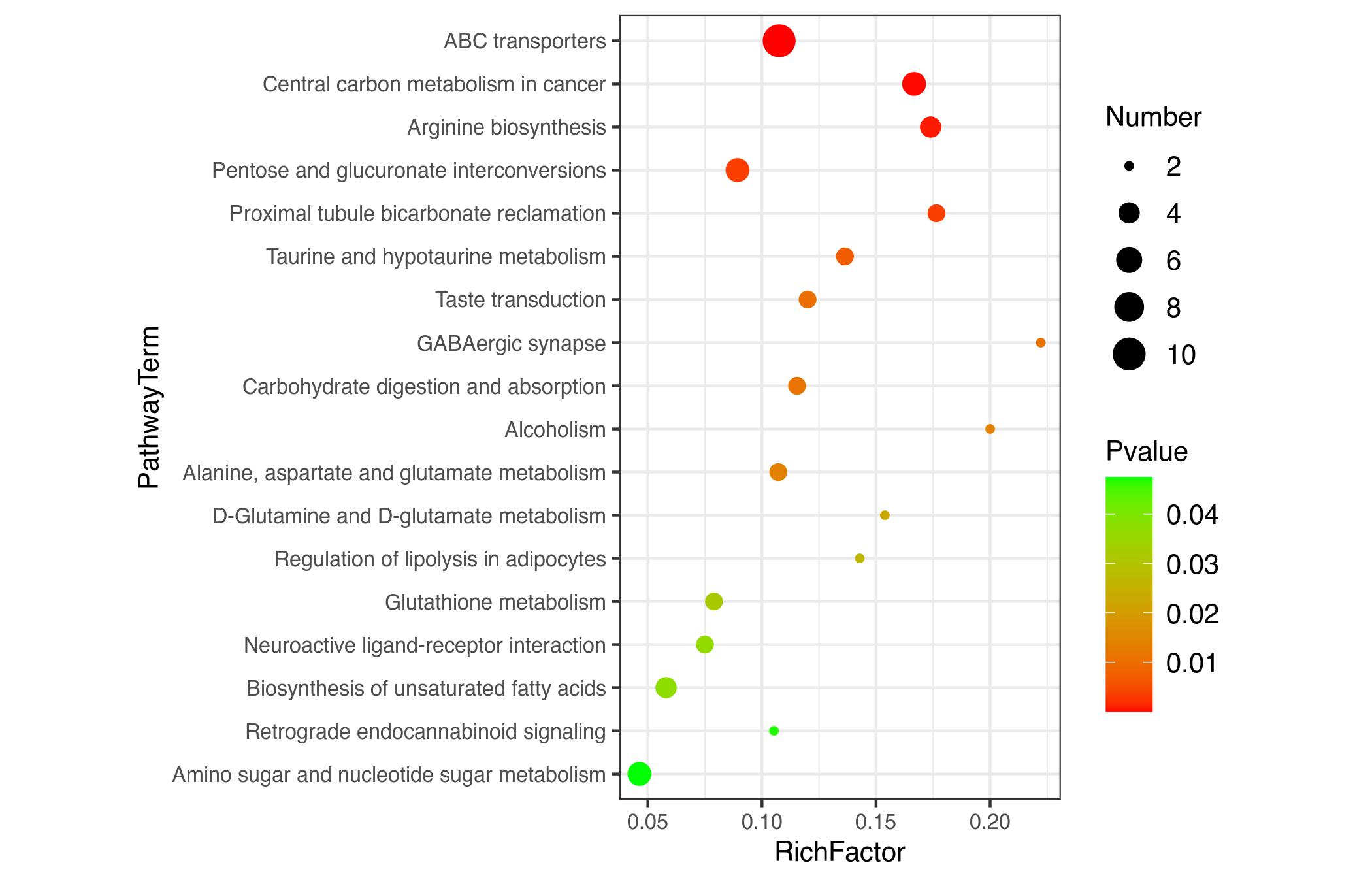
**

**Figure S3|**Enrichment analysis of the GC&LC-MS/MS metabolite in feces samples of lung cancer and healthy controls.**(A)**ADC patients and healthy controls.**(B)**SCC patients and healthy controls.**(C)**ADC and SCC patients.


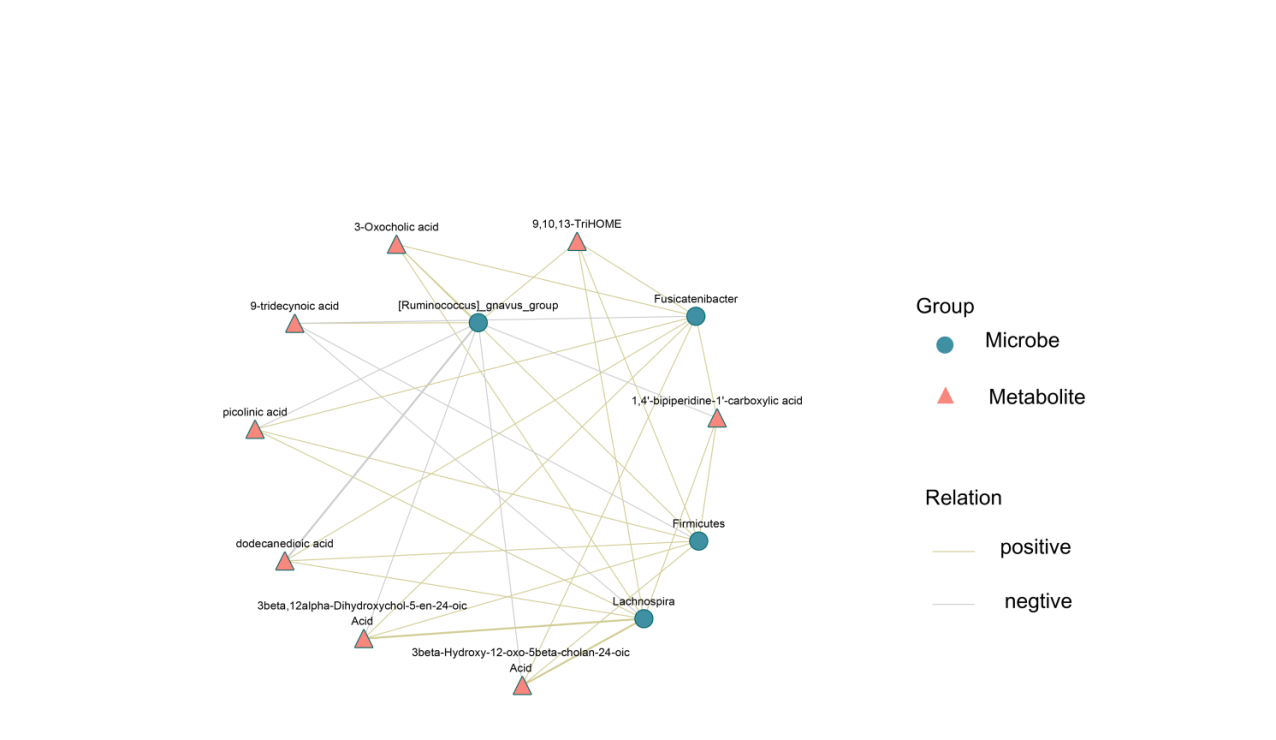


**Figure S4|**Multiomics Analysis Revealed Microbiota-Metabolite Interactions of LC.The co-occurrence network graph showed the main microbiota-metabolite correlations.
