# Supplementary material for: Structure of gut microbiota and characteristics of fecal metabolites in patients with lung cancer
Source: Front Cell Infect Microbiol. 2023 Jul 27;13:1170326. doi: 10.3389/fcimb.2023.1170326 (PMC10415071; doi:10.3389/fcimb.2023.1170326)
Supplement: Supplementary file 2 [file Table_1.docx]

Supplementary Material

# Supplementary Tables

Supplementary Table S1.The information of top five gut metabolites of LC patients

| **Compound_Name** | **RT(min)** | **m/z** | **log2FC** | **VIP** | **Super Class** | **Class** | **Score** | **FC** | **P-value** |
| --- | --- | --- | --- | --- | --- | --- | --- | --- | --- |
| **Quinic acid** | 22.8 | 345.2 | -2.4 | 2.7 |  |  | 84.7 | 0.18 | 0.000 |
| **3-Hydroxybenzoic acid** | 18.1 | 267.1 | -1.3 | 2.1 | Benzenoids | Benzene and substituted derivatives | 79.7 | 0.40 | 0.000 |
| **1-Methylhydantoin** | 11.6 | 171.1 | -1.5 | 2.4 | Organoheterocyclic compounds | Azolines | 84.1 | 0.35 | 0.001 |
| **3,4-Dihydroxybenzeneacetic acid** | 22.3 | 384.2 | -3.2 | 3.3 | Benzenoids | Phenols | 78.6 | 0.11 | 0.003 |
| **3,4-Dihydroxyhydrocinnamic acid** | 24.6 | 179.1 | -3.8 | 2.5 | Phenylpropanoids and polyketides | Phenylpropanoic acids | 96.9 | 0.07 | 0.009 |

RT: Retention Time;m/z: Mass-to-Charge Ratio; log2FC: log2 Fold Change

Supplementary Table S2.The information of top three gut metabolites of ADC patients

| **Compound_Name** | **RT(min)** | **m/z** | **log2FC** | **VIP** | **Super Class** | **Class** | **Score** | **FC** | **P-value** |
| --- | --- | --- | --- | --- | --- | --- | --- | --- | --- |
| Tyramine | 16.7 | 174.1 | 2.3 | 2.6 |  |  | 90.6 | 4.80 | 0.045 |
| 1,7-Dimethyluric acid | 3.6 | 195.1 | -3.2 | 3.9 | Organoheterocyclic compounds | Imidazopyrimidines | 52.6 | 0.11 | 0.015 |
| Stigmasterol | 29.0 | 125.1 | -2.8 | 2.6 |  |  | 77.1 | 0.14 | 0.03 |
| 3-Hydroxybenzoic acid | 18.1 | 267.1 | -3.2 | 1.1 | Benzenoids | Benzene and substitutedderivatives | 79.7 | 0.54 | 0.03 |

RT: Retention Time;m/z: Mass-to-Charge Ratio; log2FC: log2 Fold Change

Supplementary Table S3.The information of top five gut metabolites of SCC patients

| **Compound_Name** | **RT(min)** | **m/z** | **log2FC** | **VIP** | **Super Class** | **Class** | **Score** | **FC** | **P-value** |
| --- | --- | --- | --- | --- | --- | --- | --- | --- | --- |
| 3β-Hydroxycholest-5-en-26-oic acid | 13.0 | 417.3 | 1.3 | 1.4 | Lipids and lipid-like molecules | Steroids and steroid derivatives | 38.7 | 2.50 | 0.002 |
| Stearic acid | 29.2 | 117.1 | 1.1 | 1.4 |  |  | 83.3 | 2.20 | 0.004 |
| Aconitic acid | 13.4 | 229.1 | -0.5 | 1.2 | Organic acids and derivatives | Carboxylic acids and derivatives | 74.1 | 0.66 | 0.000 |
| Behenic acid | 32.9 | 397.4 | -0.5 | 1.1 | Lipids and lipid-like molecules | Fatty Acyls | 73.4 | 0.69 | 0.000 |
| Glucose-6-phosphate | 29.9 | 404.2 | -3.3 | 2.3 | Organic oxygen compounds | Organooxygen compounds | 66.6 | 0.10 | 0.002 |

RT: Retention Time;m/z: Mass-to-Charge Ratio; log2FC: log2 Fold Change

Supplementary Table S4.The information of top five gut metabolites of ADC vs SCC patients

| **Compound_Name** | **RT(min)** | **m/z** | **log2FC** | **VIP** | **Super Class** | **Class** | **Score** | **FC** | **P-value** |
| --- | --- | --- | --- | --- | --- | --- | --- | --- | --- |
| D-glucose | 25.2 | 204.1 | -2.4 | 2.7 | Organic oxygen compounds | Organooxygen compounds | 84.7 | 12.5 | 0.000 |
| Maltotriose | 24.8 | 204.1 | -1.3 | 1.3 |  |  | 89.4 | 1.10 | 0.000 |
| Stearic acid | 29.2 | 117.1 | -1.5 | 1.8 |  |  | 83.3 | 0.30 | 0.000 |
| Udp-n-acetylglucosamine | 12.22 | 12.2 | -2.2 | 1.8 | Benzenoids | Phenols | 79.9 | 0.11 | 0.000 |
| Palmitic acid | 26.5 | 117.1 | -2.3 | 2.0 |  |  | 99.6 | 0.21 | 0.000 |

RT: Retention Time;m/z: Mass-to-Charge Ratio; log2FC: log2 Fold Change
